# Prevalence of anemia and its associated factors among children under five years of age attending at Guguftu health center, South Wollo, Northeast Ethiopia

**DOI:** 10.1371/journal.pone.0218961

**Published:** 2019-07-05

**Authors:** Angesom Gebreweld, Neima Ali, Radiya Ali, Temesgen Fisha

**Affiliations:** 1 Department of Medical Laboratory Science, College of Medicine and Health Science, Wollo University, Dessie, Ethiopia; 2 Department of Laboratory, Dessie Referral Hospital, Dessie, Ethiopia; University of Ghana, GHANA

## Abstract

**Background:**

Anemia during childhood adversely affects mental, physical and social development of the children. This study is aimed to assess the prevalence of anemia and its associated factors among children less than five years of age in Guguftu, South Wollo, Northeast Ethiopia.

**Method:**

A cross-sectional health facility based study was conducted among 404 children aged 6 to 59 months. Systematic random sampling technique was used to include the participants. Pretested and structured questionnaires were used to collect socioeconomic and demographic characteristics of the family and child. Data on nutritional status, capillary blood and stool samples were collected from each child. Multivariate logistic regression was used to calculate adjusted odds ratios and the corresponding 95% confidence intervals.

**Result:**

The overall prevalence of anemia was 41.1% (95% CI;36.6% - 45.8%). Of the anemic under five children, 112 (67.5%) had mild anemia, 52(31.3%) had moderate anemia, and 2(1.2%) had severe anemia. Children who were in the age group of 6–11(AOR = 4.52; 95% CI: 1.67–12.34) and 12–23 (AOR = 2.79; 95% CI: 1.04–7.51) months, living in an urban (AOR = 1.83; 95% CI: 1.05–3.18), with no formal education mothers (AOR = 7.05; 95% CI: 2.93–17.01) and primary education mothers (AOR = 3.26; 95% CI: 1.29–8.24), with a family monthly income of <750 ETB(AOR = 5.19; 95% CI: 1.24–21.75) and 750–1500 ETB(AOR = 5.89; 95% CI: 1.45–23.98), with early (<6 months) introduction of complementary foods (AOR = 3.53; 95% CI: 1.23–10.18), Underweight (AOR = 2.11; 95% CI: 1.21–3.69) were more likely to become anemic.

**Conclusion:**

This study has revealed that the prevalence of anemia in children less than five years is high and a severe public health problem in the study area. Therefore, the policymakers should make a strategy that can reduce poverty and increase the awareness of women on breastfeeding, nutrition, and other associated factors to reduce anemia.

## Introduction

Anemia can be defined as a reduction in hemoglobin (Hb) concentration, hematocrit, or a number of red blood cells per litter below the reference interval for healthy individuals of similar age, sex, and race, under similar environmental conditions[1]. According to the World Health Organization (WHO), for under five children, the threshold Hb level for being anemic is less than 110 g/l[2].

Anemia is a global public health problem which affects 1.62 billion (24.8%) people worldwide. It occurs at all stages of the life cycle but is more prevalent in pre-school aged children (under five years). Globally, 293.1 million (47.4%) under five year's children are anemic and 67.6% of these children live in Africa [3, 4]. In Ethiopia, 57% of children age 6–59 months was anemic according to the Ethiopian 2016 Demographic and Health Survey (EDHS)report[5].

Several factors contribute to the occurrence of anemia and nearly half of (43%) the anemia cases in childhood are due to iron deficiency [6]. The deficiency may result from inadequate dietary intake of iron, malabsorption of iron, an increased iron demand during rapid growth in children and chronic blood loss. Other causes of anemia include folate and vitamin B12 and A deficiencies, Malaria, intestinal helminths, viral infections, chronic disease, hemoglobinopathies, hemolysis, and bone marrow disorders[7–10]. Different studies also claimed that factors such as age, sex, residence, early initiation of complementary food, under-nutrition, maternal health status, maternal education, and poor socioeconomic status are significantly associated with anemia [11–13].

Anemia during childhood adversely affects mental, physical and social development of the children in short- and long-term outcome; it causes abnormalities of immune function, poor motor and cognitive development, poor school performance, and reduced work productivity in the life of the children, thereby decreasing earning potentials and negatively affect national economic growth. [14–17]. Anemia is also an important cause of morbidity and mortality in African children where resources to determine the underlying etiology remain poor [18].

Even though the national and regional prevalence of anemia in under five years children are available in Ethiopia [5], data on the magnitude of anemia and its risk factors in specific settings are scarce. Studying the specific etiology and prevalence of anemia in a given setting and population group is very important to prevent or treat anemia [2]. Therefore, this study is aimed to assess the prevalence of anemia and its associated factors among children under five years of age in Guguftu, South Wollo, Northeast Ethiopia.

## Materials and methods

### Study design, period and setting

A cross-sectional health facility based study was conducted to assess the prevalence and associated risk factors of anemia among children aged 6–59 months attending at Guguftu health center from November 2017 to February 2018. Guguftu health center is located in Guguftu town in Dessie zuria woreda of South Wollo, Amhara regional state, Ethiopia. Guguftu town is 433 km far from Addis Ababa, the capital city of Ethiopia and 43 km from Dessie. The town lies between latitude11 degree North 08”47and longitude 39 degrees East 38''55 and founded at 3,975 meters above sea level. Most of the people live depend on agriculture, others depending on trade. The health center provides medical service to a catchment population of 32,000. The estimated number of children aged under five in the general population during the study period were 2257. The health center has under five outpatient department and other departments. On average 10–15 children visited under five outpatient department on daily bases.

### Population

A total of 404 under-five children were included from the children's outpatient department of Guguftu health center. The sample size was calculated using single proportion formula: n = $(z_{\alpha /2}{)}^{2}\frac{P(1-p)}{d2}$ with an assumption of a proportion of anemia among under-five children of 50% since there is no study conducted at facility level in the area or in the region. The assumptions also include 95% confidence interval and 5% marginal error. Then, a 5% non-response rate was added, the final sample size was 404. Systematic random sampling technique was used to select the study participants. The average number of children visited under five outpatient department in the preceding three months was 858. K was calculated by dividing the total number of children visiting under five outpatient department in the last three months by the sample size (K = 858/404 = 2.12). Every second children visiting under five outpatient department during study period was selected for assessment. All children aged 6–59 months who attend children’s outpatient department of Guguftu health center and their guardian or parents consented to participate in the study were included. Children who lost blood by traumatic injury or surgery, taking iron and Vitamin-A supplement during the last 3 months, and those who were taking antihelmintic drugs in 3 months were excluded from the study.

### Data collection procedure

Pretested and structured questionnaires were used to collect socioeconomic and demographic characteristics of the family and child, feeding practice and other risk factors by interviewing mother/caregivers of the child. The questionnaire was adapted from previous similar literatures.

The questionnaire was prepared in English and translated in to Amharic language and then back to English to ensure its consistency. The questionnaire was pretested in Dessie health center that was not included in the actual study area on 10% of the sample size. Based on the pilot study result, certain revisions were made for the questionnaire prior to the actual study. The interview was conducted by two trained clinical nurses in the Amharic language.

Data on nutritional status were collected by measuring the weight and height of children under age 5 during the health center visit based on WHO recommendations[19]. The length was measured for children aged 6–23 months in a recumbent position and standing height was measured for children aged 24–59 months using the measuring board. The weight of the children was measured by a Salter scale. The children were without shoes on and wearing a minimum of clothes. In addition, mid-upper arm circumference (MUAC) tape was used to measure mid-upper arm circumference of the children. Each measurement was collected twice and the mean value of the two measurements was recorded on the questionnaire. Anthropometric assessment (Height-for-Age, Weight for- Height, and Weight-for-Age) was done using the WHO Anthro software, version 3.2.2 (WHO Anthro 2009, Geneva, Switzerland).Each of the three measurements was expressed in standard deviation (SD) units of *Z*-score from the median of WHO-2006, standard population. Children whose height-for-age Z-score <-2 SD from the median of the reference population were considered stunted, weight-for-height *z*-score <−2 SD were considered wasted, and weight-for-age Z-score<−2 SD were classified as underweight[19].

Capillary blood samples were collected from each child to determine hemoglobin concentration using HemoCue Hb 201 analyzer. HemoCue cuvettes are required for the analyzer. The Hb-201+ cuvettes contain a sodium deoxycholate dried reagent that lyses red blood cells to release free Hb and form a stable azidemethemoglobin which is detected at 570 nm and 880 nm. One drop of capillary blood was carefully collected in a microcuvette from a finger prick (heel prick in the case of children age 6–11 months) after the first drop of blood was wiped off with cotton wool. The filled microcuvette was loaded in the cuvette holder of calibrated HemoCue Hb201analyser and after few seconds the hemoglobin measurement displayed. Then the results were recorded on the questionnaire. The HaemoCue method has been shown to be comparable in both accuracy and precision to the International Council for Standardization in Haematology (ICSH) reference method (cyanmethemoglobin method) for the photometric determination of Hb. The HemoCue Hb 201 analyzer has an internal electronic self test every time the analyzer is turned on, it will automatically verify the measurement performance. This test is performed at regular intervals if the analyzer remains switched on. In addition, the performance of the meter was checked on the daily bases by using control standard to increase test reliability. The test was performed by an experienced medical laboratory technologist. Standard operating procedure and manufacturers instruction were strictly followed[20, 21]. Strict aseptic technique and a separate lancet for each child were employed.

Based on WHO cut-off values, Children with Hb level <110 g/L were considered anemic. Anemic children were further categorized as children with mild anemia, moderate anemia and severe anemia which corresponds to Hb value 100–109 g/l, 70–99 g/l, and lower than 70 g/l respectively.

A clean plastic container marked with an identification number was distributed to collect a stool sample from each under-five child. Then, stool wet mount smear was prepared using saline and/or iodine solution for direct microscopic identification of intestinal parasites within 30 minutes of sample collection. The direct smear was examined first by 10x and then 40x objective for detection of helminthes eggs, larvae and protozoan parasites by experienced medical laboratory technologist.

### Data analysis procedures

All collected data were entered in to “EpiInfo version 3.1” and exported to SPSS version 20.0 statistical software for analysis. Normally distributed and continuous variables expressed as mean ± SD, and non-normally distributed variables were presented as medians (quartiles 25 and 75%). Chi-square (×^2^) test was used to compare proportions. Multivariate logistic regression was used to calculate adjusted odds ratios (OR) and the corresponding 95% confidence intervals (CI). A p-value < 0.05 was used to indicate statistical significance.

### Ethical considerations

Ethical approval was obtained from Wollo University Department of Medical Laboratory Science; a letter of support was written to Dessie health center to conduct the pre-test and to Guguftu health center to conduct the study. The study participant’s parent or guardian was informed about the purpose of the study and written informed consent was obtained before the questionnaire was administered and blood and stool samples were collected from the participant. To ensure confidentiality, participants’ data were linked to a code number. Any abnormal test results of participants were communicated to the concerned body in the Health Center.

## Result

### Characteristics of study participants

A total of 404 under-five children participated in the study from November 2017 to February 2018.The mean age of the children was 23.1 months with an SD of +/-14.4 (median: 20 months, range: 6 to 58 months). More than half of the children were below two years of age 225 (55.7%) and female 222 (55.0%). Nearly three fourth, 295 (73.0%) of the children came from the rural part of the study area. Majority 210 (52.0%) of caregivers/mothers of the children were aged between 18 and 27, 241(59.6) had no formal education, and 306(75.7%) were Housewife by occupation with mean (±SD) mothers/caregivers age of 27.1 (± 5.3) years. Majority 302 (74.8%) of the mothers had one child aged under five years, and 313 (77.5%) of the studied children had first birth order. The median (interquartile range) monthly income of the families of the studied children was 800 (600–1050) Ethiopian Birr (ETB), and 234 (57.9%) had a monthly income between 750 and 1500 ETB (Table 1).

**Table 1:** Socio-demographic characteristics of children under five years and mothers attending Gugufitu Health Center, Northeast Ethiopia, (*n* = 404).

Characteristics		Frequencies	Percent (%)
Child sex	Male	182	45.0
	Female	222	55.0
Child age	6–11 months	114	28.2
	12–23 months	111	27.5
	24–35 months	82	20.3
	36–47 months	50	12.4
	48–59 months	47	11.6
Residence	Urban	111	27.5
	Rural	293	72.5
Mothers age	18–27 years	210	52.0
	28–38 years	187	46.3
	39–40 years	7	1.7
Mothers education	Non-formal	241	59.6
	Primary	109	27.0
	≥ Secondary	54	13.4
Mothers occupation	Housewife	306	75.7
	Small-scale business	16	4.0
	Government employee	3	0.7
	Farmer	79	19.6
Family’s monthly income	< 750 ETB	147	36.4
	750–1500 ETB	234	57.9
	> 1500 ETB	23	5.7
Birth order	1^st^	313	77.4
	2^nd^	54	13.4
	3^rd^	25	6.2
	4^th^ and above	12	3.0
Number of children aged <5 years	0	14	3.5
	1	302	74.7
	≥2	88	21.8

ETB = Ethiopian birr

Regarding nutritional status, more than half 211(52.3%) of the children were underweight, 146(36.1%) were wasted, and 214(53%) were stunted. Only 30 (7.4%) of the children had started complementary foods before 6 months of their age. The stool investigations showed that 4.2% of children suffered from worm infestations (Table 2).

**Table 2:** Anthropometric status and Intestinal helminthes infection of children under five years attending at Gugufitu Health Center, Northeast Ethiopia, (*n* = 404).

Characteristics		Frequencies	Percent (%)
Introduction of complementary foods	< 6 months	30	7.4
	> 6 months	374	92.6
Underweight	Not underweight	193	47.8
	moderate	96	23.8
	severe	115	28.5
Wasted	Not wasted	258	63.9
	moderate	63	15.6
	severe	83	20.5
Stunted	Not stunted	190	47.0
	moderate	79	19.6
	severe	135	33.4
Low MUAC	Yes	297	73.5
	No	107	26.5
Intestinal parasite	positive	17	4.2
	negative	387	95.8

MUAC = mid-upper arm circumference

### Prevalence of anemia among under-five children

**Fig 1:**
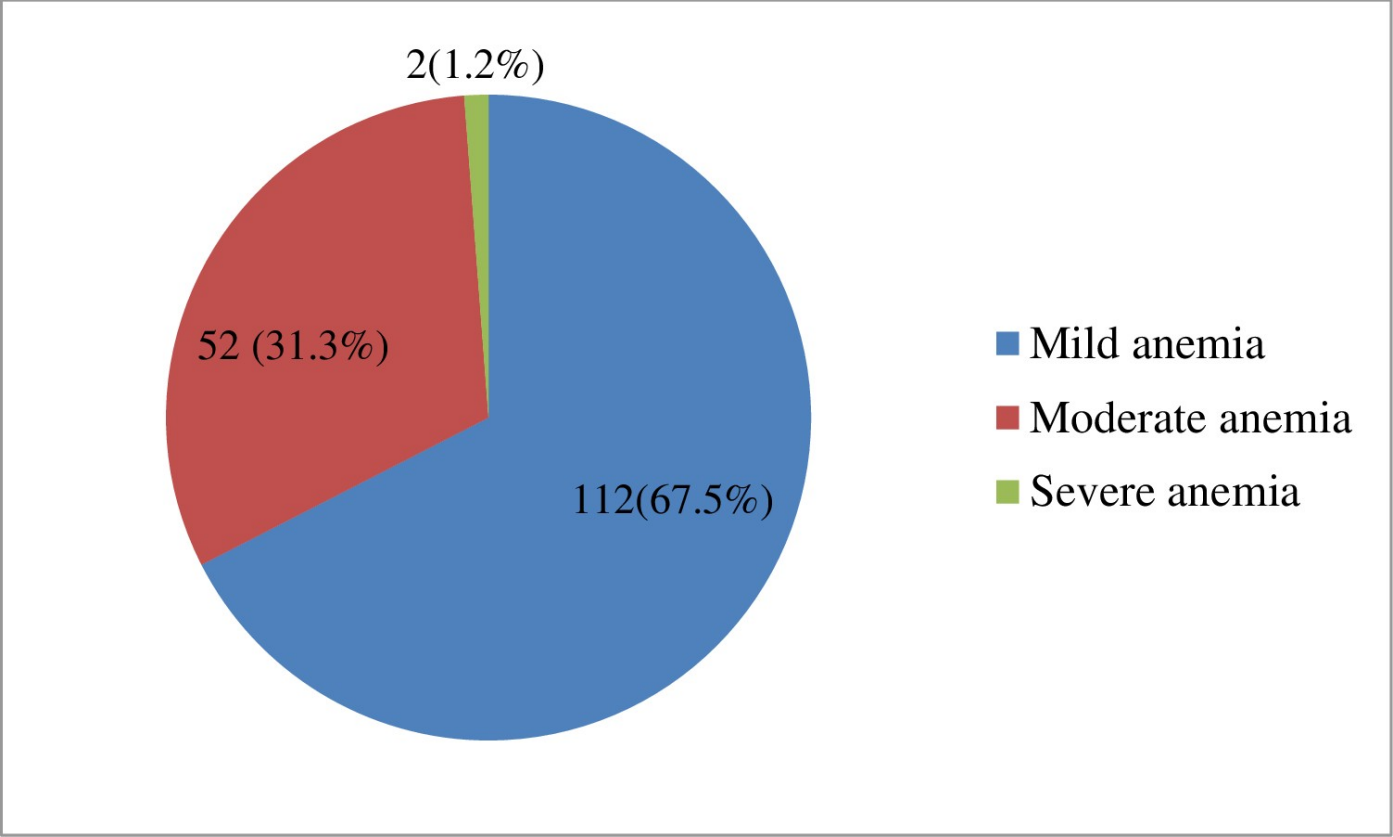
Distribution of anemia by severity among the anemic children under five years attending Gugufitu Health Center, Northeast Ethiopia, (*n* = 166).

In this study, the overall prevalence of anemia was 41.1% (95% CI;36.6% - 45.8%). The mean ± SD hemoglobin concentration among the study participants was 11.16 ± 1.53 g/dl. Of the anemic under-five children, 112(67.5%) had mild anemia, 52(31.3%) had moderate anemia, and 2(1.2%) had severe anemia (Fig 1).

**Fig 2:**
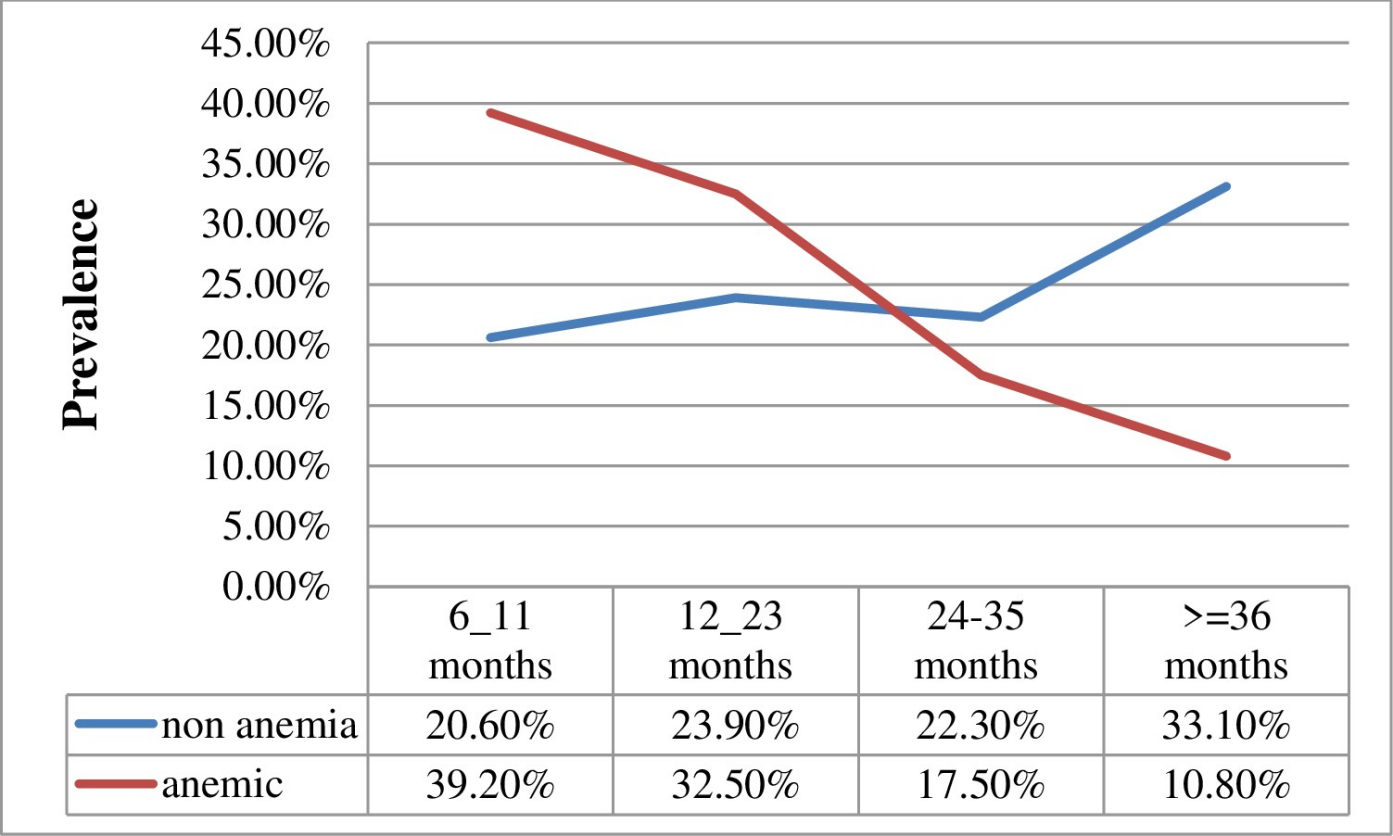
Distribution of anemia prevalence by age in children under five years attending Gugufitu Health Center, Northeast Ethiopia.

No difference was observed in prevalence between males and females (39.6% vs. 42.3%; *P* = 0.572). Among the age groups, the highest prevalence was recorded in the age group of 6–11 months (57.0%) and it gradually decreases as the age of the children increased (Fig 2).

High prevalence of anemia was observed in children who were urban dwellers (49.5%), in children whose mothers aged > = 39(57.1%) and had no formal education (48.1%). In addition, the high prevalence rate of anemia was found among children with a family monthly income of less than 750 ETB (47.6%), early (<6 months) introduction of complementary foods (73.3%), low MAUC (48.1%), and underweight (50.2%) (Table 3).

**Table 3:** Prevalence of anemia among children under five years attending Gugufitu Health Center, Northeast Ethiopia, (*n* = 404).

Characteristics		Anemia		*P* value*
		No n(%)	Yes n(%)	
Child sex	Male	110(60.4)	72(39.6)	0.572
	Female	128(57.7)	94(42.3)	
Child age	6–11 months	49(43.0)	65(57)	<0.001
	12–23 months	57(51.4)	54(48.6)	
	24–35 months	53(64.6)	29(35.4)	
	36–47 months	42(84.0)	8(16.0)	
	48–59 months	37(78.7)	10(21.3)	
Residence	Urban	56(50.5)	55(49.5)	0.033
	Rural	182(62.1)	111(37.9)	
Mothers age	18–27 years	105(50)	105(50)	0.001
	28–38 years	130(69.5)	57(30.5)	
	39–40 years	3(42.9)	4(57.1)	
Mothers education	Non-formal	125(51.9)	116 (48.1)	<0.001
	Primary	69(63.3)	40(36.7)	
	≥ Secondary	44(81.5)	10(18.5)	
Mothers occupation	Housewife	174(56.9)	132(43.1)	0.290
	Small-scale business	10(62.5)	6(37.5)	
	Gov employee	3(100.0)	0(0.0)	
	Farmer	51(64.6)	28(35.4)	
Family’s monthly income	< 750 ETB	77(52.4)	70(47.6)	0.006
	750–1500 ETB	141(60.3)	93(39.7)	
	> 1500 ETB	20(87.0)	3(13.0)	
Birth order	1^st^	178(56.9)	135(43.1)	0.481
	2^nd^	35(64.8)	19(35.2)	
	3^rd^	17(68.0)	8(32.0)	
	4^th^ and above	8(66.7)	4(33.3)	
Number of children aged <5 years	0	4(28.6)	10(71.4)	0.058
	1	183(60.6)	119(39.4)	
	≥2	51(58.0)	37(42.0)	
Introduction of complementary foods	< 6 months	8(26.7)	22(73.3)	<0.001
	> 6 months	230(61.5)	144(38.5)	
Underweight	No	133(68.9)	60(31.1)	<0.001
	Yes	105(49.8)	106(50.2)	
Wasted	No	159(61.6)	99(38.4)	0.171
	Yes	79(54.1)	67(45.9)	
Stunted	No	111(58.4)	79(41.6)	0.930
	Yes	127(59.3)	87(40.7)	
Low MUAC	Yes	154(51.9)	143(48.1)	<0.001
	No	84(78.5)	23(21.5)	
Intestinal parasite	No	231(59.7)	156(40.3)	0.129
	Yes	7(41.2)	10(58.8)	

ETB = Ethiopian birr; MUAC: mid-upper arm circumference

*significant at p < 0.05

### Factors associated with anemia

After including risk factor variables with a *P*< 0.30 on the bivariate analysis (Child age, Residence, Mothers age, Mothers education, Mothers occupation, Family’s monthly income, Number of children aged <5 years, Introduction of complementary foods, Underweight, Wasted, Low MUAC, and Intestinal parasite),multivariate logistic regression analysis was performed to identify the independent association of each risk factor with anemia. Multivariate analysis showed that children in the age group of 6–11 and 12–23 months were 4.5 times (AOR = 4.52; 95% CI: 1.67–12.34) and 2.8 times (AOR = 2.79; 95% CI: 1.04–7.51) more likely to be anemic than children in the age range of 48–59 months, respectively. Children living in an urban area were 1.8 times (AOR = 1.83; 95% CI: 1.05–3.18) more likely to be anemic than those living in a rural area. Children with illiterate (or no formal education) mothers, and with primary education were 7 times (AOR = 7.05; 95% CI: 2.93–17.01) and 3.3 times (AOR = 3.26; 95% CI: 1.29–8.24) more likely to be anemic than children with mother of secondary and above education level. Children with a family monthly income of <750 ETB, and 750–1500 ETB were 5.2 times (AOR = 5.19; 95% CI: 1.24–21.75) and 5.9 times (AOR = 5.89; 95% CI: 1.45–23.98) more likely to be anemic than children with a family monthly income of >1500 ETB. Children with early (<6 months) introduction of complementary foods was 3.5 times (AOR = 3.53; 95% CI: 1.23–10.18) more likely to be anemic than children with timely (>6 months) initiation of complementary foods. Similarly, Underweight children were 2.1times (AOR = 2.11; 95% CI: 1.21–3.69) more likely to be anemic than children with normal weight (Table 4).

**Table 4:** Factors associated with anemia among children under five years attending Gugufitu Health Center, Northeast Ethiopia, (*n* = 404).

Variable	Anemia		Crude OR (95% CI)	Adjusted OR (95%CI)
	Yes, n (%)	No, n (%)		
Age				
6–11 months	65 (57.0)	49 (43.0)	**4.91 (2.23–10.82)** *	**4.52 (1.67–12.34)** *
12–23 months	54 (48.6)	57 (51.4)	**3.51 (1.59–7.74)** *	**2.79 (1.04–7.51)** *
24–35 months	29 (35.4)	53 (64.6)	2.03 (0.88–4.65)	1.66 (0.63–4.39)
36–47 months	8 (16.0)	42 (84.0)	0.71 (0.25–1.97)	0.43 (0.12–1.48)
48–59 months	10 (21.3)	37 (78.7)	1.00	1.00
Residence				
Urban	55 (49.5)	56 (50.5)	**1.61 (1.04–2.50)** *	**1.83 (1.05–3.18)** *
Rural	111 (37.9)	182 (62.1)	1.00	1.00
Mothers educational status				
Illiterate/No formal	116 (48.1)	125 (51.9)	**4.08 (1.97–8.49)** *	**7.05 (2.93–17.01)** *
Primary	40 (36.7)	69 (63.3)	**2.55 (1.16–5.62)** *	**3.26 (1.29–8.24)** *
≥ Secondary	10 (18.5)	44 (81.5)	1.00	1.00
Monthly income				
<750 ETB	70 (47.6)	77 (52.4)	**6.06 (1.73–21.28)** *	**5.19 (1.24–21.75)** *
750–1500 ETB	93 (39.7)	141 (60.3)	**4.40 (1.27–15.22)** *	**5.89 (1.45–23.98)** *
>1500 ETB	3 (13.0)	20 (87.0)	1.00	1.00
Introduction of complementary foods				
< 6 months	22 (73.3)	8 (26.7)	**4.39 (1.91–10.13)** *	**3.53 (1.23–10.18)** *
> 6 months	144 (38.5)	230 (61.5)	1.00	1.00
Underweight				
No	60(31.1)	133(68.9)	1.00	1.00
Yes	106(50.2)	105(49.8)	**2.24(1.49–3.36)** *	**2.11(1.21–3.69)** *

OR = odds ratio, CI = confidence interval, 1 = reference

*statistically significant association (p<0.05)

## Discussion

This study assessed the prevalence and associated factors of anemia among children aged 6 to 59 months in Guguftu, Northeast Ethiopia. The overall prevalence of anemia was 41.1% (95% CI;36.6% - 45.8%), this finding was similar to 2016 Ethiopian DHS prevalence reported for the Amhara Region (42%) [5]and study conducted in Jimma, Ethiopia (46%) [22]. However, the result of the present study is lower than studies conducted in Nepal (49.5%)[23], South-East Nigeria (49.2%)[24],Hohoe municipality and Volta Regional Hospital of Ghana(47.5% &55.0%)[25, 26], and Limpopo Province, South Africa (75.0%)[27].The difference in the prevalence might be due to variation in the study design, sampling techniques, and sample size. The difference might also be due to variation in the geographical location of the study participants or due to variation in socio-demographic characteristics or socioeconomic status of parents in the areas.

Regarding the levels of anemic status, in this study, the majority of the anemic children had mild anemia (67.5%) followed by moderate anemia (31.3%) and severe anemia (1.2%). This finding is in agreement with studies conducted in Nepal [23], Hohoe municipality, Ghana[25], and India [28]. However, our result is deviated from EDHS 2016 report [5] and study in Volta Regional Hospital of Ghana[26], which showed high moderate anemia.

In this study, there was a higher prevalence of anemia among children under two years old and it decreased as the age of the children increased. This finding is supported by other studies conducted in Ethiopia [5, 29, 30] and other developing countries [25, 31, 32].This might be due to high iron demands associated with rapid growth rate and erythropoiesis, diets poor in bio-available iron, and low maternal iron reserve during pregnancy [33].

Similar to study reports in South-East Nigeria [24] and the Volta Regional Hospital of Ghana [26], the present study found that sex difference did not show association with anemia. However, other studies found a higher prevalence of anemia among boys than girls [11, 29], and also in girls than boys [27]. This inconsistency may be explained by the social norms in differential intake of iron-rich foods between genders; however, subsequent studies are required to better understand this complex issue.

The present study found an association between the levels of maternal education and anemia which is in agreement with other studies [11, 32, 34–37]. Children of mothers with low educational levels were 3.3–7 times more likely to be anemic than children of a mother with secondary and above education level. This may be explained by the fact that education enhances the mother's knowledge needed for their children’s health and an appropriate feeding practice, which help to improve their children nutritional status. However, this finding is inconsistent with the study conducted in Northwest Ethiopia [38]. The present study also found that children with low family income were 5.2–5.9 times more likely to be anemic than those with high family income. This finding was similar to studies conducted in other parts of the country and countries, which reported that children from poor families were at risk of anemia compared to their counterparts [11, 13, 30, 32, 36].A possible explanation for the high prevalence of anemia might be that families with low income are less likely to purchase nutrient-rich foods (like iron, vitamins, and etc), secure food availability, and afford health service during illness for their children. The higher prevalence of anemia among children with less educated mothers and low-income families indicates that anemia is a marker of socioeconomic disadvantage. [39, 40].

The observed higher prevalence of anemia among children with early (<6 months) introduction of complementary foods was consistent with previous studies [11, 41]. Complementing breast milk before 6 months of age reduces the bioavailability of iron by up to 80%; the early introductions of complementary foods like cow's milk interfering in the absorption of iron in the breast milk because cow's milk has excess protein and minerals notably calcium and most digestive enzymes are inadequate at this age. In addition, early initiation of complementary feeding expose to microbial pathogens due to contamination and resulting high risk of diarrheal diseases, thereby malabsorption [5, 42].However, this finding is in contrary to study in Sri Lanka which showed that children who were exclusively breastfed for 6 months or more were more likely to be anemic than children who were exclusively breastfed for less than 6 months[37].

Nutritional status also associated with anemia among children aged 6–59 months. In this study, Underweight children were 2.1times more likely to be anemic than children with normal weight. This finding is similar to a study conducted in Northern Ethiopia[30] and Brazil [43]. Usually, the causes of anemia and underweight (malnutrition) are similar and aggravated by poverty and food insecurity. Food insecurity affects the nutritional status of children by compromising the quantity and quality of dietary intake, which contributes for development of anemia [6, 40].

One of the limitations of this study is the cross-sectional nature of the study design; it does not reveal causal links between independent variables and anemia. Due to constraint of resource, we were unable to measure serum ferritin concentration, soluble transferrin receptor concentration, folate levels, vitamin B12 levels, thalassemia, and G6PD deficiency; which could have helped in finding the causes of anemia. The other limitation is that this study is conducted at a health center; hence, further community based studies should be conducted to have findings more representing the whole population. Despite the limitations, we have determined the magnitude of anemia and identified important factors associated with anemia among children aged 6–59 months in Guguftu health center.

## Conclusions

This study has revealed that the prevalence of anemia in children less than five years is high and a severe public health problem in the study area. Age, residence, family income, maternal education, an introduction of complementary foods, and nutritional status were the factors significantly associated with anemia. Therefore, the policymakers should make a strategy that can reduce poverty and increase the awareness of women on breastfeeding, nutrition, and other associated factors to reduce anemia.

## Supporting information

S1 File Questionnaire used for data collection. (DOCX)

S1 Dataset (XLSX)
