# Supplementary material for: Prevalence of anemia and its associated factors among children under five years of age attending at Guguftu health center, South Wollo, Northeast Ethiopia
Source: PLoS One. 2019 Jul 5;14(7):e0218961. doi: 10.1371/journal.pone.0218961 (PMC6611584; doi:10.1371/journal.pone.0218961)
Supplement: S1 File — (DOCX) [file pone.0218961.s001.docx]

## English Version Questionnaire

Questionnaire to assess the prevalence of anemia and associated factors among under five children attending Guguftu health center ,South Wollo, Northeast Ethiopia.

Questionnaires ID.No______________ Address______________

Name of health facility____________________ Date of interview____________

Time started_________________ Time finished______________

Participant code _________________

**Part I: Questions on socio-demographic, economic characteristics and** **anthropometric measurement of the child**

1. Age of child (months) ______________
2. Age of mother (in years) _______________
3. Sex

| 1. Male | 1. Female |
| --- | --- |

1. Residence

| 1. Urban | 1. Rural |
| --- | --- |

1. Mother’s education

| 1. Illiterate | 1. Non formal education |
| --- | --- |
| 1. Primary | 1. Secondary |
| 1. College and above |  |

1. Mother’s occupation

| 1. Housewife | 1. Small scale business |
| --- | --- |
| 1. Employed | 1. Farmer |

1. Monthly income of the household (in Ethiopian Birr) ________________
2. Introduction of complementary foods at the Age of:

| 1. ≤ 6 month | 1. > 6 months |
| --- | --- |

1. Number of children <5 years ________________
2. Birth order of the child _____________________
3. Birth interval of the child (in month/year) ______________
4. Current weight of the child (in kilogram) ________________
5. Current height of the child (in centimeter [cm]) ______________
6. Mid upper arm circumference (MUAC) (in cm)____________________

Name of the interviewer _____________________________________

Signature _________________ Date ______________

**Part II: Laboratory result**

1. Stool examination for intestinal parasite

| 1. No O/P seen | 2. Positive (list name of parasite )   1. ______________ 2. _______________ 3. ________________ |
| --- | --- |

1. Blood film examination for malaria

| 1. No Hemoparasite seen | 1. P. f |
| --- | --- |
| 1. P. v | 1. Mixed |

1. Hemoglobin level _________________ g/dl or g/l

Name of the Laboratory professional_______________________

Signature _________________ Date ______________

**Amharic version questionnaire**

የመጠይቁ መ.ቁ…………….. አድራሻ…….......... የጤናተቋሙስም .................. ቃለመጠይቅ የተደረገበት ቀን ………………….. የተጀመረበት ሰዓት…………… ያለቀበት ሰዓት…………… የተሳታፊ ኮድ ……….

**ክፍል አንድ፡ሥነ-ሕዝብ፣ኢኮኖሚ እና የህጻኑ አንትሮፖሜትሪ የተመለከቱ ጥያቄዎች**

1. የህጻኑ ዕድሜ (በ ወር)­­­­………………………….
2. የእናት እድሜ (በ ዓመት): ………………
3. የህጻኑ ጾታ

| 1. ወንድ | 1. ሴት |
| --- | --- |

1. የመኖርያ ቦታ

| 1. ከተማ | 1. ገጠር |
| --- | --- |

1. የእናትየዉ የትምህርት ደረጃ

| 1. ያልተማረች 2. የመሰረተ ትምህርት 3. 1^ኛ^ደረጃ (1-8ኛ) | 1. 2^ኛ^ደረጃ(9-12ኛ) 2. ኮለጅ እና ከዛ በላይ |
| --- | --- |

1. የእናትየዉ ሰራ

| 1. የቤት እመቤት | 1. ነጋዴ |
| --- | --- |
| 1. መንግስት ሰራተኛ | 1. ገበሬ |

1. የቤተሰብ ወርሃዊ ገቢ ስንት ነው?: …………… ብር
2. ህጻኑ ተጨማሪ ምግብ የጀመረበት ግዜ መቼ ነዉ?

| 1. ከ 6 ወር በፊት | 1. ከ 6 ወር በኃላ |
| --- | --- |

1. ከ 5 ዓመት በታች ህጻናት ብዛት ………………
2. ህጻኑ ስንተኛ ልጅ ነዉ?.....................
3. ህጻኑ ከታላቁ የስንት ወር/ዓመት ልዩነት አላቸዉ?.......................
4. የህጻኑ የአሁን ክብደት በ ኪ.ግ……………..
5. የህጻኑ የአሁን ቁመት በ ሴ.ሜ………………
6. የክንድ ዙርያ ልኬታ (MUAC) (በ ሴ.ሜ)……………..

የጠያቂዉ ሰም……………………

ፌርማ………………………………………. ቀን፡……………………

**ክፍል ሁለት፡ የላቦራቶሪ ዉጤት**

1. የሰገራ ምርመራ ለ አንጀት ጥገኛ ተሀዋስያን ዉጤት

| 1. ምንም ኦቫ/ፓራሳይት አልተገኘም | 2. ፖዘቲቭ (የተገኘዉ/ኙ ፓራሳይት ግለጽ)   1. ______________ 2. _______________ 3. ________________ |
| --- | --- |
|  |  |

1. የዎባ ምርመራ ዉጤት
2. ምንም ሄሞፓራሳይት አልተገኘም
3. ፕ.ፋልሲፐረም
4. ፕ.ቫይቫክስ
5. ሚክሰድ
6. የሄሞግሎቢን ዉጤት _________________ ግ/ዴሊ or ግ/ሊ

የላቦራቶሪ ባለሞያዉ ስም………………………………………

ፌርማ………………………….. ቀን……………………
